# A Comparative Study of Anomaly Detection Techniques for Smart City Wireless Sensor Networks

**DOI:** 10.3390/s16060868

**Published:** 2016-06-13

**Authors:** Victor Garcia-Font, Carles Garrigues, Helena Rifà-Pous

**Affiliations:** Internet Interdisciplinary Institute (IN3), IT, Multimedia and Telecommunications Department, Universitat Oberta de Catalunya, Rambla del Poblenou 156, 08018 Barcelona, Spain

**Keywords:** anomaly detection, information security, outlier detection, smart cities, support vector machines, wireless sensor networks

## Abstract

In many countries around the world, smart cities are becoming a reality. These cities contribute to improving citizens’ quality of life by providing services that are normally based on data extracted from wireless sensor networks (WSN) and other elements of the Internet of Things. Additionally, public administration uses these smart city data to increase its efficiency, to reduce costs and to provide additional services. However, the information received at smart city data centers is not always accurate, because WSNs are sometimes prone to error and are exposed to physical and computer attacks. In this article, we use real data from the smart city of Barcelona to simulate WSNs and implement typical attacks. Then, we compare frequently used anomaly detection techniques to disclose these attacks. We evaluate the algorithms under different requirements on the available network status information. As a result of this study, we conclude that one-class Support Vector Machines is the most appropriate technique. We achieve a true positive rate at least 56% higher than the rates achieved with the other compared techniques in a scenario with a maximum false positive rate of 5% and a 26% higher in a scenario with a false positive rate of 15%.

## 1. Introduction

In the last few years, cities around the world are building new smart city systems, which rely on advanced communication protocols and the latest technology to improve their operational structure and to acquire a data-driven management perspective. In order to gather urban information, smart cities use elements of the Internet of Things (IoT), such as mobile phones, RFID cards and wireless sensor networks (WSNs). The data collected by these devices is used in a plethora of applications. For example, traffic monitoring sensors are used to control traffic lights [1] and wireless meters are installed in pipes to monitor leaks and ruptures [2]. Moreover, this data gives city managers and other stakeholders the opportunity to plan future facilities using a better picture of citizens’ behavior and the real use of the current infrastructures.

The clear benefits provided by smart city technology have stimulated many cities to devote a considerable part of their innovation efforts to develop their concept of a smart city. This has caused a significant and fast increase in the number of WSN deployments on the streets, which has resulted in the emergence of new applications with many different technologies, solutions, requirements, *etc*.

However, this accelerated deployment of smart city technology has often resulted in leaving security aside as a secondary issue. For instance, some studies [3,4] have proven that traffic control systems can be manipulated in real deployments in the United States due to the lack of cryptographic and authentication systems in the sensors and, in general, because of a systematic lack of security consciousness.

Moreover, in order to rapidly deploy WSNs and smart city technology, cities have taken advantage of services procured from external providers. Nevertheless, outsourcing public services has also raised security-related concerns [5].

The impact of these outsourcing policies on security can mainly be attributed to two key factors: the loss of control over network devices and the lack of visibility over the potential security problems affecting these devices. Indeed, public administration usually outsources not only the implementation and deployment of their WSNs, but also the administration thereof. In this way, security countermeasures and system logs are exclusively operated by external providers. Although service providers are contractually obliged to ensure certain levels of security, in practice, smart city administrators cannot determine the extent to which the received data is precise and accurate. In fact, the Royal Academy of Engineering has identified data quality as one of the six major barriers to effectively optimize smart infrastructures [6].

In order to mitigate the control and visibility problems affecting the data quality of the smart city data collection systems, in this article we propose the use of anomaly detection algorithms. These algorithms use the network status information received at the city data centers to determine if the behavior of the networks is reliable.

However, in the smart city context, performing anomaly analysis using network status information is a challenging problem, because most WSNs provide different amounts of status information. Most smart cities combine multiple deployments of WSNs, each one with different objectives and requirements, and usually installed by different external providers. This results in many different WSN technologies being used at the same time in different parts of the city, and each providing different amounts of status information to local council administrators.

Taking all these issues into consideration, we present a comparative study of different anomaly detection algorithms and we analyze their behavior, taking into account the minimum quantity of network status information that they require to accomplish their goal. This will undoubtedly help administrations to implement efficient anomaly detection techniques and to determine the minimum status information that should be required from external providers in future WSN deployments.

In order to compare the detection capabilities of the different algorithms, we have simulated WSNs based on real data gathered in actual WSN deployments in the smart city of Barcelona, and we have also generated anomalies simulating well-known attacks against WSNs. To assess the different algorithms, we have computed the most widespread metrics in these types of comparative studies. As far as we know, this is the first comparative study in this field from the smart city perspective.

The rest of this paper is structured as follows: Section 2 contains related work and Section 3 contains background. The simulation and the experimental procedure are explained in Section 4. Section 5 contains the results of this study. Finally, Section 6 concludes the paper.

## 2. Related Work

In this section, we first review the types of attacks in WSNs and the countermeasures proposed in the literature (Section 2.1). Second, we introduce anomaly detection analysis (Section 2.2).

### 2.1. Attacks in WSNs

The limited computational and energetic constraints of nodes are an obstacle to applying conventional computer network security countermeasures to WSNs. Furthermore, in these networks, nodes become more vulnerable when they are placed in unprotected environments like streets. In these circumstances, attackers can easily capture nodes, access confidential information in their memory (e.g., cryptographic keys) and reprogram their behavior. It is also common that attackers benefit from the wireless nature of the communications to intercept the messages or to obstruct frequency bands to impede the proper reception of some packets. Surveys such as [7,8] summarize the most popular attacks on WSNs against authenticity, confidentiality, integrity and availability, and also present some solutions.

In order to counter the effects of these attacks and increase the reliability of these networks, numerous studies have proposed the use of fault tolerance mechanisms. These mechanisms are intended to add multipath routing capabilities to the networks, thus ensuring connectivity between nodes even when a link or node fails. A good example of such mechanisms is [9], where the authors propose two algorithms to repair broken paths in mesh topologies recomputing only small parts of the topological structure where the broken links are located. This efficient route recovery mechanism consumes less energy and uses a smaller amount of control packets than other similar algorithms. On the other hand, in [10], the authors propose a secure mechanism based on path redundancy to offer a fault and intrusion tolerant routing scheme, which outperforms similar mechanisms in mean time to failure, energy consumption computation overhead and resiliency.

Even though these mechanisms are highly recommendable for WSN providers to increase their network reliability levels, they are not applicable on a global scale in a smart city. As previously mentioned, most smart cities combine multiple WSN technologies and configurations, some of which are incompatible with the definition of alternative paths (e.g., in networks with a star or a tree topology).

On the other hand, a large number of studies have focused on preventing attacks on WSNs against authenticity, confidentiality, integrity and availability. Surveys such as [7,8] summarize the most popular attacks and also present some solutions.

Problems related to authentication and confidentiality are normally tackled with cryptographic solutions. The first WSN nodes were designed with minimum processing power, which makes legacy systems based on these networks incapable of running any cryptographic algorithm. However, in the last few years, manufacturers have developed more powerful nodes and new protocols have been designed to take into account these cryptographic requirements. For example, the most popular communication protocols for WSN, e.g., 802.15.4 and ZigBee, include different security modes based on symmetric cryptography [11]. Asymmetric cryptography has also been proposed in [12] through an SSL protocol for WSNs.

Cryptography is also a mechanism to avoid integrity attacks. Checksums and Message Authentication Codes (MACs) are the usual countermeasures to impede unnoticed modifications of packets in transit. The destination node of an altered packet discards it if the received packet and the code generated by the message integrity mechanism do not match. However, integrity attacks are hardly noticed by city administrators since most WSNs do not send information to the base station indicating the reasons why packets were dropped. Thereby, the traces of this type of attack can be assimilated to the traces of attacks against data availability.

Attacks against availability normally focus on breaking communication in certain areas and depleting node batteries. Representative attacks against this principle are selective forwarding and jamming. In a selective forwarding attack, the attackers capture some nodes of the network with routing activities. Then, the captured routers forward only certain packets from other nodes. Selective forwarding can affect several services if it is performed in a gateway or another node shared by multiple WSNs. In a jamming attack, attackers send a high power signal to disrupt wireless transmissions from legitimate nodes. This type of attack not only affects the target network, but also other networks using the same frequency band.

Although the literature proposes solutions to avoid attacks against data availability, they are not always effective or applicable. For instance, frequency hopping spread spectrum [13] is used to avoid certain types of jamming attacks by constantly changing the transmission channel within the frequency band of the protocol. However, jammer devices currently available on the market can jam all the channels used by several protocols at the same time. Attacks against availability are very hazardous because they slow down networks, drop a large amount of packets, deplete node batteries, can compromise several city services and there are no effective countermeasures against them. The best mitigation approach against these type of attacks is a good detection strategy. Therefore, this article focuses on discovering data availability attacks.

### 2.2. Anomaly Detection

Within the research field of intrusion detection, two types of techniques can be distinguished: misuse detection and anomaly detection. Whereas the former seeks traces left by the attackers in the security data (e.g., system logs), the latter analyzes the normal behavior of the system and points out unusual changes.

Intrusion detection techniques looking for misuses rely on an extensive database of attack signatures. An attack signature is a sequence of actions that are normally recorded in a security log. The signature can be used to identify an attacker’s attempt to exploit a known network, operating system or application vulnerability. Alarms are raised when the detection system discovers a sequence of events that matches any of the signatures [14]. The main advantage of this type of detection is the low rate of false positives. In the context of WSNs in smart cities, signature-based detection is useful to identify attacks targeting networks with regular behavior (e.g., environmental sensors sending readings every day at the same hour) or highly reliable services. Simple rules can be created in these two cases to trigger alerts when the expected readings are not received or when a certain number of packets are lost. Nonetheless, many smart city services do not follow a regular pattern and WSN is an unreliable technology, where some packets are occasionally not delivered.

Alternatively, intrusion detection techniques looking for anomalies are able to identify changes in the system that do not match the normal behavior. Anomaly detection has been widely used in many application domains (see a survey on anomaly detection techniques in [15]). The most common techniques fall into the scope of statistics, clustering and machine learning. Depending on the types of samples necessary to process the data, these techniques are divided into supervised, semi-supervised or unsupervised.

Supervised techniques require a training dataset with labels indicating the category of each sample (e.g., ‘no attack’, ‘jamming’ or ‘selective forwarding’). Then, a model is generated to classify new unlabeled samples into one of the defined categories. Semi-supervised techniques require a training dataset with samples of a single category in order to create a model that classifies new samples as belonging to that category or not. Finally, unsupervised techniques do not require labeled training data and are capable of dividing a dataset into various subsets without a previously learnt model.

Depending on the characteristics of the specific scenario and on the requirements of the application, some algorithms perform better than others. For instance, the authors of [16] compare several unsupervised approaches based on local outlier factor, near neighbors, Mahalanobis distance and Support Vector Machines to detect intrusions in conventional computer networks. Their experiments show that the local outlier factor approach is the most adequate in this context.

Regarding anomaly detection in Intrusion Detection Systems (IDS) for WSNs, Xie *et al.* [17] surveys the most popular techniques. Generally, the nodes that contain IDS components gather and/or analyze network status data concerning anomalous operation activities of their neighbors. When this occurs, the nodes trigger an alarm at the base station. Anomaly detection techniques have been applied in multiple applications related to WSNs.

As an example, the authors of [18] use geostatistics and time-series analysis to detect outliers in readings of meteorological sensors. The authors select temporal and spatial real-data-based outlier detection (TSOD) as the most appropriate technique in this context. In their experiments, the authors claim that TSOD has a high performance and it is able to identify all the outliers with a low false positive rate around 3%. However, these techniques are only applicable to certain scenarios in which there exists a spatio-temporal correlation and the WSN is dense enough.

Other studies focus on anomaly detection applied to single sensors. As an example, Su *et al.* [19] proposes a two-phased algorithm. In the first phase, the algorithm seeks temporal anomalies with one-class Support Vector Machines (OC-SVM) and, in the second phase, the algorithm reduces false positives and classifies the anomalies with a supervised K-Nearest Neighbor approach (KNN). For the first phase, the authors compare OC-SVM with other techniques (*i.e.*, logistic regression, random forest, linear SVC, complexity invariant distance based KNN and Euclidean distance based KNN). The authors conclude that OC-SVM outperforms the other techniques achieving a 96% detection rate in their experiments.

In [20] Mahalanobis distance is used to detect insider attacks with high detection accuracy and robustness (*i.e.*, the false positive rate stays low even though the number of outlying sensors increases). Some authors claim that anomaly detection techniques based on the distance to the neighbors should not be used in WSN due to high computational complexity [21]. Nevertheless, from the point of view of smart city administrators, these techniques can be considered because anomaly analysis can be computed in data centers using powerful computers.

In [22], the authors use one-class quarter-sphere Support Vector Machines (QSSVM) in two new anomaly detection algorithms: LADS and LADQS. These algorithms are suitable to run in constrained nodes due to their low computational complexity. Moreover, their experiments show a high performance, e.g., 95% true positive rate and a false positive rate below 10%.

The authors of [23] use an improved Autoregressive Integrated Moving Average (ARIMA) model to predict anomalies in WSN through network traffic analysis in the nodes. The experiments in the article show an accuracy higher than 96% and a false positive rate lower than 3%.

Although some of the previously mentioned anomaly detection techniques and IDS perform well in detecting attacks, they are not a generalizable solution in an heterogeneous context such as the smart city. This is due to the fact that, on the one hand, some techniques excessively depend on the context of the WSN. On the other hand, IDS are normally designed ad-hoc to be embedded in some or all the nodes of specific WSNs. Therefore, IDS can only be considered as a first protection mechanism to be implemented by WSN providers for their specific networks. From the centralized perspective of the smart city administration, the techniques must not require access to the WSN nodes nor knowledge of the specific technology used by each external provider.

## 3. Background

The following sections describe the anomaly detection techniques compared in this article. In the smart city context, it is not possible to assume that data from all the possible attack categories is available in the training dataset (*i.e.*, some attacks are unknown until new vulnerabilities are disclosed). Accordingly, supervised techniques are not suitable in this context. Regarding semi-supervised and unsupervised techniques, it is also necessary not to base the attack detection on the previous knowledge of the problem. For instance, some statistical techniques assume specific distributions of the data. However, this is unknown in many smart city services and sometimes the data behavior is variable depending on the time of day, the season of the year, the weather conditions, *etc*.

Moreover, smart city services usually provide multivariate data, where several features define each sample. These features are the basic information used by the detection algorithms to identify outliers within a dataset. In this context, samples with attacks have to be marked as outliers. Therefore, in this article, several multivariate semi-supervised and unsupervised techniques (that do not require previous knowledge about the scenario) are compared in order to identify attack samples. The compared techniques are the most frequently used methods in the literature for this purpose, and they are based on Mahalanobis distance, local outlier factor, hierarchical clustering and OC-SVM.

Apart from these techniques, we have considered other more recent methods from the area of machine learning. For example, algorithms based on random forests [24] have been used successfully in many scenarios of different domains, but their popularity nowadays has not reached the levels of SVM. Another family of algorithms that is certainly worth considering is deep learning. In this regard, recent advances show that this is a very promising field. As an example, algorithms based on deep belief networks [25] convolutional neural networks [26] or recursive neural networks [27] have been used successfully in several scenarios to improve the performance obtained with previous techniques. In the area of anomaly detection, additionally, the authors of [28] have used deep learning in combination with other techniques to identify outliers, and they have obtained promising detection results.

However, the use of deep learning for anomaly detection is a research field that is still too unripe. Deep learning techniques, in general, require costly training processes, which is something easily attainable in fields such as computer vision, speech recognition, *etc*. Nevertheless, in the case of smart city WSNs, obtaining large training datasets is much more complex due to their dynamic nature. This dynamic behaviour involves retraining the models generated by the machine learning algorithms frequently, thus making it difficult to work with huge training datasets and applying the deep learning techniques successfully.

### 3.1. Mahalanobis Distance

Mahalanobis distance measures the number of standard deviations that an observation is from the mean of a distribution. This measure can be used to detect outliers in multivariate data, because outlier observations do not have normal values in one or more dimensions. Hodge *et al.* [29] surveys outlier detection methodologies and compares Mahalanobis distances with other proximity-based outlier detection techniques.

### 3.2. Local Outlier Factor

Local Outlier Factor (LOF) is a degree measuring the isolation of a point in a vector space with respect to its neighbors [30]. In order to compute this degree of isolation, LOF is based on the concepts of reachability distance and local reachability density (lrd). The reachability distance between two points *p* and *q* is the maximum value between the distance between *p* and *q* and the farthest distance between *q* and its *k* nearest neighbors. The lrd for the point *p* is the inverse of the average reachability distance between *p* and its *k* neighbors. Finally, the LOF computes the average ratio of the lrd of *p* with the lrd of its *k* neighbors. LOF values smaller than 1 indicate high densities, LOF values greater than 1 indicate low densities and LOF values close to 1 indicate average density spaces. Outliers are considered to be in low density regions.

In [30], the authors of LOF suggest a lower and an upper bound for the *k* value. The lower bound for *k* can be considered as the minimum amount of nearby points that can mark out a more isolated nearby point as an outlier. It is considered good practice to select a *k* higher than 10 to remove unwanted statistical fluctuations. On the other hand, the upper bound for *k* indicates the maximum number of nearby points that can potentially be considered outliers. A group of k−1 or less nearby points require other points in the vector space to have *k* points to compute the LOF. This implies that the LOF values for the points in the group increases and becomes similar to the LOF of the isolated points. Therefore, either some isolated points are considered normal or the points in the group are considered outliers. In their experiments, the authors of LOF indicate that the algorithm performs well selecting values of *k* between 10 and 20.

### 3.3. Hierarchical Clustering

Hierarchical clustering is a type of analysis that aims at partitioning a dataset in groups of data (*i.e.*, clusters) according to a similarity measure and creating a tree-based structure that eases the anomaly analysis. This clustering analysis is performed using two types of approaches: top-down or bottom-up [31]. In this work we focus on agglomerative hierarchical clustering, which is a bottom-up approach, where initially each sample of the dataset falls in a different cluster and, in each step of the algorithm, two clusters are selected according to a similarity measure and combined in a new cluster. This process ends when there is only one cluster that includes all the samples. A common similarity measure can be computed using the Euclidean distance in Ward’s method [32]. With this method, two clusters with the minimum average distance from any sample in one cluster to any sample in the other cluster are merged in each step.

Agglomerative hierarchical clustering can be used to compute outlier ranking factors for the samples in the dataset. Outliers are theoretically more dissimilar to other observations and they should be more resistant to be merged in a new cluster. Thereby, various methods have been proposed to obtain the outlier factors with this type of clustering, such as *linear*, *sigmoid* or *sizeDiff* [33]. In this work we use *sizeDiff*.

### 3.4. Support Vector Machines

Classification techniques based on Support Vector Machines (SVM) have proven to be effective in several contexts related to intrusion detection [34,35]. Basically, classification techniques based on machine learning require two steps. First, a dataset is used to train a learning model. Then, the trained model is used to classify new data samples. Several features define each sample of the datasets. The SVM classification process represents the training dataset in a *n*-dimensional vector space, *n* being the number of features of the training data. Then, it defines a hyperplane (*i.e.*, a n−1 dimensional plane) that separates (with a maximum margin) the samples from the different classes. The Support Vectors (SV) are the subset of training samples that are near the hyperplane and that define it. Finally, the hyperplane acts as a frontier to classify other samples.

In this article we use one-class Support Vector Machines (OC-SVM), which are a special case of semi-supervised SVMs that do not require attack labeled data. OC-SVMs build a frontier to classify new samples as normal or outlier. In SVM, different types of kernel functions are available to build the most adequate hyperplane for each application. In this work, we use a Radial Basis Function (RBF) kernel, which can learn complex regions [15].

## 4. Simulation and Anomaly Detection Analysis

In this section, we first mention some challenges in testing security solutions for smart cities (Section 4.1). Second, we explain how we overcome these challenges in this work and we briefly describe the procedure that we use to evaluate different anomaly detection algorithms (Section 4.2). The other sections contain information about the different steps in the analysis: the data collection (Section 4.3), the simulation (Section 4.4), the feature selection (Section 4.5) and the anomaly analysis (Section 4.6).

### 4.1. Smart City Security Simulation Challenges

Smart cities can be considered as very heterogeneous scenarios, where many technologies, applications and different suppliers coexist. Thus, the implementation of software simulators that realistically reflect the complexity of the smart city WSNs is very complicated. In recent years, simulators have been used to test new protocols and to assess the security techniques that protect simple WSNs in very specific contexts [36,37]. Among the most popular WSN simulators, we can find: OMNET++ [38], Castalia [39], Cooja [40] and NS-2 [41].

From a technological perspective, replicating WSN configurations from different providers to simulate several smart city scenarios is a very arduous task. This is motivated by the extensive variety of existing hardware on the market and the wide availability of communications protocols for WSNs. Furthermore, although some of the previously mentioned simulators implement realistic signal propagation algorithms, none does so with a model that can take into account complex urban components, such as walls, traffic, *etc*. Moreover, simulators also lack realistic event generation engines to reproduce the dynamics of the citizens and the other elements interacting with the urban WSN. For example, Castalia offers multiple distribution functions to simulate the events sensed by the sensors. Nonetheless, selecting the appropriate distribution and modeling the appropriate behavior for the different applications is complex and can lead to unrealistic conclusions. Recently, the authors of CupCarbon [42] proposed a simulator for an easy integration of WSNs and elements of the IoT in smart cities. However, this simulator, which is intended as a supplement to other simulators, is still immature and it does not implement all the layers of the communication stack.

Performing simulations to test security components poses additional difficulties. On the one hand, reproducing computer attacks requires a high technological expertise and a high investment in manpower. On the other hand, many attacks exploit unknown vulnerabilities and, therefore, they are not a priory replicable in controlled simulated environments.

Moreover, the testing of security issues in controlled contexts using real hardware is complicated in a smart city. For example, many WSN applications in the cities cannot be easily deployed at a similar scale in a realistic testbed because they would require an infrastructure as big and dynamic as a city. In addition, attack tests in operational WSN are generally incompatible with some application requirements (e.g., 24/7 availability) and can be detrimental to third parties (e.g., jamming attacks to ZigBee can also provoke interferences to WiFi users).

Therefore, it is necessary to combine the large amount of data that is already gathered by smart city providers on the streets with the use of existing simulators to evaluate the consequences of computer attacks and to determine the most appropriate intrusion detection techniques and the appropriate security procedures to resolve these issues.

### 4.2. Experimental Procedure

In order to overcome the barriers discussed in the previous section, we use real data from deployed services in Barcelona to feed a WSN simulator that will generate data following realistic patterns. Running experiments in the simulator provides the flexibility to test different communication protocols and network configurations and it is also a safe way to execute computer attacks. This section presents how we use this mechanism to collect a smart city WSN dataset with and without attacks, and how we compare four anomaly detection techniques to detect intrusions based on Mahalanobis distance, local outlier factor, hierarchical clustering and one-class Support Vector Machines. We also compare the performance of these techniques under different amounts of available network status information, taking into account three different levels of permitted false positive rates.

**Figure 1:**
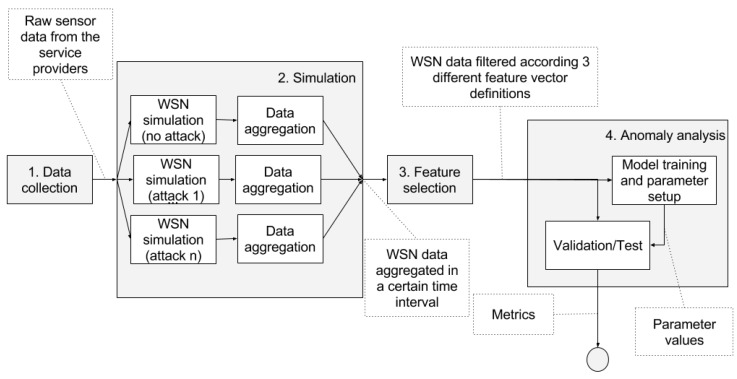
Pipeline of the simulation and the experimental process.

The pipeline in Figure 1 shows a general picture of the complete process of the analysis. This process consists of the following steps:

1. Data collection: we gather raw sound data over a period of 14 days from the streets of Barcelona (Section 4.3).
2. Simulation (Section 4.4):
  (a) We use the raw data in a simulator to generate WSN data with comprehensive information about all the communication layers. The simulation is executed multiple times (one time without including any attack and one time for each of the attacks) resulting in a dataset containing samples with and without attacks.
  (b) The simulation data is aggregated in time intervals.
3. Feature selection: we filter the features of the dataset according to those available at the simulated WSN (Section 4.5). As previously stated, the main goal of this article includes minimizing the amount of network status information required to detect anomalies. Thus, we select the features taking into account the simulated availability of network status information.
4. Anomaly analysis (Section 4.6). We select one of the available detection techniques and we proceed with the following sub-steps:
  (a) Training phase: we train a model or we setup the parameters required by the detection technique.
  (b) Validation/Test phase: we test the performance of the technique to distinguish between the samples that were generated with and without attacks. At this stage, we compute the metrics to compare the different techniques.

We repeat steps three and four with three different feature sets for each of the four detection techniques that we compare in this study. We discuss the results taking into account the different situations in Section 5.

### 4.3. Data Collection

**Figure 2:**
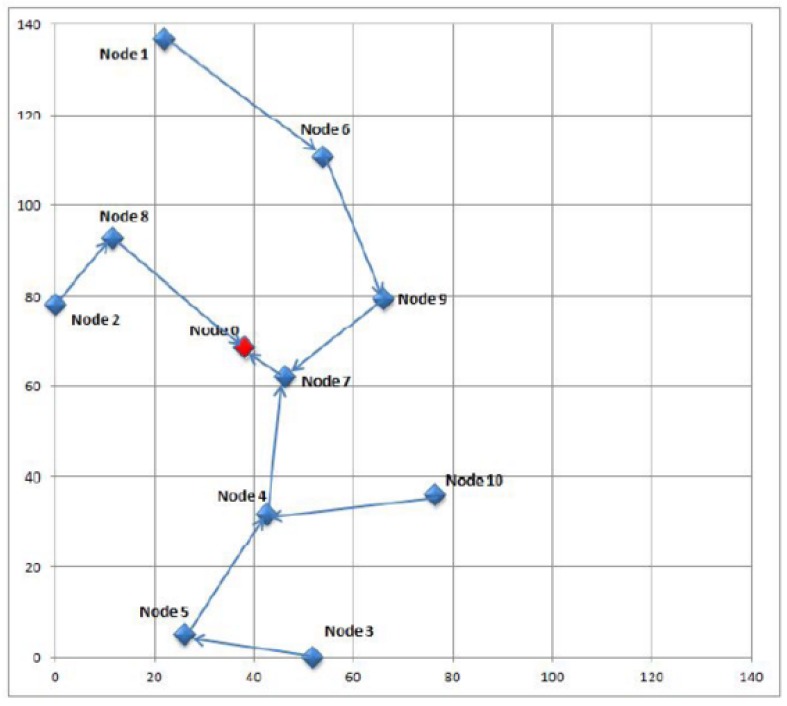
Schema and topology of the simulated WSN. The layout of the sensor nodes (*i.e.*, nodes 1-10) reproduces the layout of real sound meters deployed in Barcelona over a 140 m × 140 m terrain. The topology and the base station (*i.e.*, node 0) location are setup ad-hoc for the simulation.

The first step in this study is the collection of real urban data. The scenario for this simulation is based on data gathered during 14 days from sound meters deployed in the city of Barcelona. The sound meters, which are installed on the streets by a service provider, send their readings to the smart city central servers. The layout of the sensor nodes is represented in Figure 2. The outcome of this first step is a dataset with raw sound data.

As Figure 2 shows, the WSN contains a reduced number of sensors belonging to a single provider, even though networks gathering data from a city service can be much more complex, involving many more nodes and several providers. In case of anomalies, however, the network should be divided in smaller sections, because this allows administrators to reduce the search for the specific compromised equipment to a smaller area with fewer nodes and less providers. It falls beyond the scope of this article and remains as future work to find an optimal procedure to divide large WSNs into smaller ones in a scalable manner.

### 4.4. Simulation

The raw data from the sound readings obtained in the previous step is used in the second step to build a realistic simulated scenario of a smart city service with Castalia 3.3 simulator [39]. This simulator can aggregate information from all the layers involved in the communication between the sensors and the base station using different configurations in a WSN. In the studied real WSN implementations, most of this network status information is currently not disclosed by service providers and, therefore, it is unavailable at the smart city data centers. Thus, in this paper we are analyzing the effects of including this information to detect attacks.

In order to use the real sound readings in the simulations, we implemented an application module [43] in Castalia that replays the exact sending behavior of the real sound devices. In this way, the simulated sensors acquire the same sending patterns as the real sensors deployed on the streets.

The simulation also takes into account that WSNs are unreliable networks in which packets can be lost even in non-attack circumstances. To mimic this behavior, Castalia’s physical and communication layers lose some packets. This circumstance is paramount in order to evaluate the detection techniques in a realistic scenario, where communications are not always perfect. Moreover, we also include two nodes from which no messages are received because of failed communication and inactivity. The simulated WSN uses the CC2420 [44] communication module, configured in TMAC [45] and follows a multihop tree topology as it can be seen in Figure 2.

In step 2, the simulation runs to generate data with and without attacks. The implemented attacks exemplify two easy ways to attack WSNs, which can also be disruptive for third party WSNs in smart cities. Moreover, the attacks cover different levels of affectation in terms of the number of compromised nodes in the network and in terms of disrupted packets. The following are the implemented attacks:

- **Constant jamming.** Attack at the physical and link layers, where the attackers send a high power signal to a legitimate node in order to avoid the correct reception of legitimate packets from other nodes. Besides disrupting application packets, this attack has also an effect on MAC protocols, because the attacker also jams control packets and occupies the channel for a long time, which disrupts the coordination among nodes and impedes other nodes from starting their transmission. We implemented this attack in three situations: near node 4 (affecting 4 nodes in the lower area of the network), near node 9 (affecting 3 nodes in the higher area of the network) and near the base station (affecting all the nodes in the network).
- **Selective forwarding.** Attack at the network layer, where the attackers have captured the base station and they drop a percentage of random packets before re-transmitting them to the smart city control center. We implemented this attack in four levels: a selective forwarding dropping 30% of the packets, 50%, 70% and 90%.

Besides simulating the WSN events in step 2 Castalia aggregates the outcome in time intervals. This outcome is mainly a set of variables containing network status information about the communication protocol for each node. For instance, the number of radio packets received with interferences during a certain period of time.

The size of the time window is a paramount parameter in the detection process of attacks concerning data availability. On the one hand, short attacks can get obscured among a plethora of data in large time windows. On the other hand, datasets in small time windows sometimes do not contain enough variability to be able to distinguish between normal and attack situations.

Having a too large or too small time window also depends on the type of monitored service. For instance, during the 14 days of the sound data gathering process, we measured in Barcelona an average of 3085 messages per hour per sensor from a parking service and 100,057 messages per hour per sensor from an electrical meter service. This implies that an attack against the electrical meters during a few minutes drops several messages and becomes easily visible, whereas the same attack against the parking sensors does not always leave traces in the data since a lack of messages from the parking sensors can be normal for several minutes. In the simulation for this article, we divide the 14 day sound data in 30 min time windows. As a result, the dataset contains 5.344 samples of eight classes (*i.e.*, one class for the 668 samples with no attack and one class for each of the seven attacks). Each sample contains information such as the number of received application packets and the battery used during the interval.

### 4.5. Feature Selection

As previously stated, the main goal of this paper is to evaluate several semi-supervised and unsupervised techniques in different situations considering different degrees of network status information availability. To achieve this goal, this status information is converted into features in a vector space, which is then explored by the anomaly detection algorithms to identify the attacks. The feature vectors extracted from the WSN data determine the set of variables included in the learning models of these algorithms. These variables are the basic knowledge to decide if each sample in the dataset contains anomalies.

In other machine learning applications, a large number of features are gathered and a feature extraction transformation (e.g., Principal Component Analysis [46]) is executed prior to classification in order to reduce the dimensionality of the vector space. However, in our context, the necessary features have to be chosen from the inception of the process. This is due to the fact that adding extra features requires computing, sending and forwarding more information from the WSN nodes, and so, would have a negative impact on the network performance and the sensors battery life. Therefore, in this article, we compare the performance of the detection algorithms, taking into account three different situations related with the available features:

- **Feature Vector 1 (FV1)**. This includes data aggregated from the minimum information that any WSN always sends (*i.e.*, the sensor readings and the timestamp). The aggregated features are: the number of application packets received at the central server and the hour of the day.
- **Feature Vector 2 (FV2)**. This includes FV1 fields plus the data extracted and aggregated from supplementary fields included in the packets (*i.e.*, the sequence number of the application packet and the battery level). The aggregated features are: the ones in FV1 and also the number of lost application packets and the consumed energy.
- **Feature Vector 3 (FV3)**. This includes FV2 fields plus data aggregated from the principal components of the WSN communication protocol in the physical, link, network and application layers. The additional features included in this feature vector per node are: the number of proper radio transmissions with and without interferences; the number of failed radio transmissions due to interference, the low sensitivity and incorrect reception state; the number of received MAC ACK and CTS.

The necessary information to build FV1 and FV2 is already available in some real WSN implementations in Barcelona, whereas the extra information required to aggregate the data to build FV3 is currently not available in any implementation. In fact, with FV3, we are evaluating the case where administrators use all the available features to train and test the models. Even though not all the features are necessarily relevant to disclose attacks, we are testing the resistance of the algorithms to increasing the vector dimensionality with non-relevant features.

The outcome of the feature selection step is the dataset from the previous step filtered according to one of the feature vector descriptions.

### 4.6. Anomaly Analysis

The anomaly analysis [43] step compares four different techniques using *R* [47]. The first technique is implemented with the *stats* [47] package and it is based on Mahalanobis distance. The second technique computes the LOF score with *DMwR* [33]. For the third technique, we compute an outlier score using agglomerative hierarchical clustering analysis according to Ward’s clustering method [32]. This score is obtained with the method *sizeDiff* through the function *outliers.ranking* in *stats*. Finally, we use the *e1071* [48] package for the fourth technique: a one-class classification with OC-SVM.

**Figure 3:**
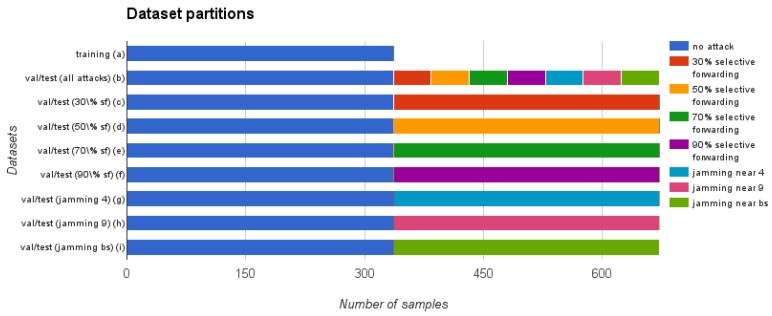
Size of the dataset partitions. The validation and test (val/test) datasets are partitioned in the same manner and contain the same number of samples of each attack type.

The anomaly analysis comprises three basic sub-steps for each of the compared techniques: the training, the validation and the test phases. In order to perform these sub-steps, first of all, our study takes the filtered dataset obtained in the feature selection step and divides it as shown in Figure 3. As this figure shows, the attack samples are not included in the training dataset (a), because the detection techniques that we use in this article are semi-supervised or unsupervised. Regarding the validation and test datasets, each of them is divided into 8 additional datasets ((b) to (i) in the figure), resulting in a total of 17 datasets (16 + 1 training dataset). As we will describe in the next section, we use these datasets to run 72 experiments to evaluate the four anomaly detection techniques proposed in Section 2.2.

Once we have these dataset partitions, basically, we will use the training dataset to tune the parameters required by the algorithms. We will use the validation dataset internally during the development of the experiments to estimate the performance of the algorithms. Finally, we will use the test dataset just once to obtain the results published in this paper. The following sections include more details about these datasets and the actions taken in the training (Section 4.6.1), validation and test phases (Section 4.6.2).

#### 4.6.1. Training Phase

The main responsibility of the training phase is to find the best parameters for the algorithms and to fit the models. We use the training dataset, which contains only samples without attacks, to perform these two tasks.

Before training the models and selecting the parameters, we first normalize and standardize the features in all the datasets (*i.e.*, substracting the mean and dividing by the standard deviation for each feature) and then we identify the features that have a zero variance in the training dataset. These features are removed from the three datasets (*i.e.*, training, validation and test). Thereby, the features that do not provide any information for the detection process are eliminated. We use the remaining features in the training dataset to train the models and to find the best parameters for the algorithms considering three different levels of false positive rate: permissive (false positive rate < 15%), restrictive (false positive rate < 10%) and very restrictive (false positive rate < 5%). From now on, we will refer to these levels as the permitted false positive rate (PFPR). We consider that a rate higher than 15% overwhelms administrators with an excessive number of false alarms.

In order to select the optimum parameters for the OC-SVM, we use grid search [49]. In this method, a grid with parameter values is exhaustively explored in order to select the values that give the best performance using the training dataset. OC-SVM requires the set up of two parameters: *ν* and *γ*. We fix the value of *ν* to the PFPR since the training dataset does not contain any samples with attacks and the *ν* value is a higher limit on the fraction of outliers in the training dataset [50]. We use grid search to find the best value for *γ* using *svm.tune* [48] configured in a 10 fold cross-validation repeated 3 times [51]. This function uses the classification error as a performance measure to decide the best value for *γ*.

Before the different detection techniques can be compared, an additional step has to be carried out, since the OC-SVM technique returns a binary value (which simply indicates if the sample is an outlier or not) and the LOF, Mahalanobis and hierarchical clustering techniques return an outlier score (in our context, outliers will be considered as attacks). Thus, the outlier score must be translated into a decision on whether the sample is considered an outlier or not. In order to do so, for each of these score-based techniques, we select a threshold score beyond which the sample is considered an outlier. This threshold score is determined as the threshold where the false positive rate in the training dataset is equal to the PFPR. The procedure is as follows:

1. The outlier score is computed for each sample in the training dataset using the corresponding detection algorithm. This results in a list *L* of scores.
2. Any sample in the training dataset identified as attacked should be considered as a false positive (FP), since this dataset does not contain any attack. Therefore, the maximum amount of allowed false positives in the training dataset is defined by the PFPR (*i.e.*, FP≤|L|*PFPR).
3. The |L|*PFPR highest score in *L* is set as the threshold.

Furthermore, LOF also requires the definition of the parameter *k*. In this article, we have determined that k=10 is a good choice following the indications of [30], as we described in Section 3.2.

#### 4.6.2. Validation and Test Phase

The validation and test datasets are used to evaluate the performance of the algorithms in 72 experiments: (1 with all the attacks together + 7 with each attack separately) x 3 feature vector definitions x 3 PFPR levels. As Figure 3 shows, these datasets contain the same number of samples and each is divided into several partitions. Dataset (b) contains half of the dataset without attacks and the other half with attacks, with equal proportion of samples from each of the seven attack types. This dataset allows us to validate and test the behavior of the detection algorithms in a general way, taking into account all the attacks. Moreover, we also create validation and test datasets (c) to (i) that only include samples of a single attack. These datasets allow us to evaluate the performance of the algorithms for each of the different attacks separately. We balance the number of samples with and without attack in each of the datasets using sampling with replacement [52].

In the validation and test phases, we use the detection algorithms to decide whether each sample has to be considered as an attack or not. Then, we count the correct identifications of attacks as true positives, the incorrect identifications of attacks as false positives, the correct identifications of no attacks as true negatives and the incorrect identifications of no attacks as false negatives. Then, we compute the metrics described below. The training and validation phases are iteratively conducted to explore the most suitable configurations of the algorithms. These configurations are then applied in the test phase to obtain the results that we show in next section.

The metrics we use in this study evaluate the detection results for the different anomaly detection techniques, taking into account the cases where the algorithms correctly detect an attack, the cases where the attacks are not detected and also the cases where the algorithms incorrectly point out an attack that has not occurred. These metrics, which have been widely used to assess IDSs and machine learning algorithms [16], are the following (see details in Table 1): the true positive rate (also known as detection rate, sensitivity or recall), which measures the percentage of attacks that have been properly detected; the false positive rate (also known as the false alarm rate), which indicates the percentage of normal samples misclassified as attacks; and the f-score, which is used as a general overview of the performance of the algorithm. This metric takes into account the number of true positives over the arithmetic average of predicted positives and real positives.

## 5. Results and Discussion

**Figure 4:**
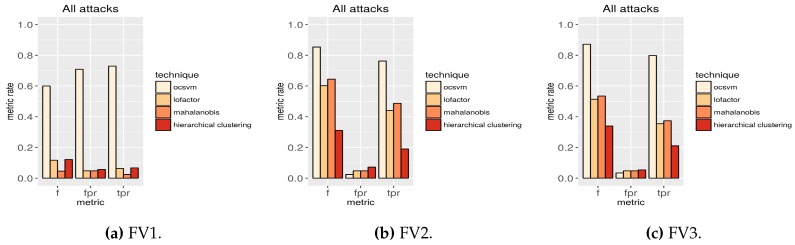
Results using the test dataset with samples of all the attacks filtering the features according to the three feature vector definitions with a very restrictive PFPR. The plots show the metrics f-score (f), the false positive rate (fpr) and the true positive rate (tpr). The captions below each plot indicate the feature vector definition used in each case.

In this section, we show the most relevant results of the 72 experiments. As we mentioned, these experiments evaluate the detection algorithms using the different feature vector definitions for the different PFPR on the test dataset partitions shown in Figure 3. Only the most important information is included in this section (Figure 4, Table 2 and Table 3). The remaining results are shown in the supplementary materials.

Filtering the datasets according to the feature vector definition FV2 and using OC-SVM is the optimal approach for attack detection in the context described in this article. Table 2 (sorted by the true positive rate column) presents the performance of the algorithms using samples with all the attack types in the test dataset (b) and filtering the features according the three feature vector definitions. The top rows in the table show that OC-SVM is the technique performing the best in terms of true positive rate and false positive rate. For all the PFPR, OC-SVM performs better than any of the other techniques. The minimum difference in the performance occurs with a permissive PFPR. In this case the true positive rate using OC-SVM is 37% higher than using LOF, 28% higher than using Mahalanobis distance and 26% higher than using hierarchical clustering. With a very restrictive PFPR (Figure 4), which is the most challenging configuration to disclose attacks, the difference in the performance is the highest. In this case the true positive rate using OC-SVM is 73% higher than using LOF, 56% higher than using Mahalanobis distance and 300% higher than using hierarchical clustering. In this last configuration, the true positive rate using OC-SVM is over 75% and the f-score over 85%.

From a theoretical point of view, the results suggest that a large amount of samples with attack lie too close to samples without attack in the vector space. Therefore, techniques based on distances (*i.e.*, Mahalanobis, LOF and hierarchical clustering) cannot distinguish between the two types of samples in many cases (specially in the most restrictive situations). However, in OC-SVM, the results suggest that the separating hyperplane resulting from the training process is closely adjusted to the data without attacks. As a result, samples with attacks lie, in most cases, outside the frontier defined by this hyperplane, even when these samples are very near to the ones without attacks.

Furthermore, as we can see in the supplementary materials, using the features defined by FV2, OC-SVM also gives the best results for all the metrics in 18 of the 21 experiments when using test datasets (c) to (i), which contain only samples of a single type of attack (*i.e.*, 7 attacks × 3 feature vector definitions). However, in three experiments (Table 3), the false positive rate exceeds the PFPR. In the experiment with 30% selective forwarding, the false positive rate exceeds the very restrictive PFPR by 6.7 percentage points and the restrictive PFPR by 1.7 percentage points. In the experiment with 50% selective forwarding, the false positive rate exceeds the very restrictive PFPR by less than 1 percentage point. Although these three experiments show the false positive rate as slightly over the PFPR, the other 18 experiments show that OC-SVM is generally the most suitable in this context.

Unlike filtering features with FV2 or FV3, when filtering is performed with FV1, datasets with and without attacks show only a slight variation. For example, as Table 4 shows, when data in the feature vectors are normalized and we compute the mean of the standard deviation among all the features, with FV1 the difference between including and excluding attacks is minimal (*i.e.*, 0.48 for the training dataset and 0.45 for the test dataset). On the other hand, this difference is larger with the other two feature vector definitions (*i.e.*, 0.39 for the training dataset and 0.60 for the test dataset with FV2). Including only features from FV1 makes attack samples and normal samples to lie very close in the vector space. Therefore, the performance of all the compared techniques is generally very poor in this case as it can be seen in Table 2. With FV1, the highest true positive rate is achieved when dealing with attacks that affect a large number of nodes (*i.e.*, 90% selective forwarding attack and jamming attack near the base station). In this case, the technique based in hierarchical clustering achieves a true positive rate around 30% if PFPR is permissive. Hence, in the scenarios where only the features in FV1 are available, none of these techniques is suitable. Therefore, we can conclude that public administrations should never allow WSN providers to supply so little network status data.

Finally, Figure 4 also shows that OC-SVM is the only technique resistant to the inclusion of too many features for the algorithms. With the extra features included in FV3, the rest of the algorithms decrease their performance. SVMs do not depend on the size of the vector space to be able to properly generalize [53]. This technique shows more resistance to high dimensionality and to the inclusion of correlated features.

Furthermore, in the scenario using the extra features included in FV3, the detection performance of the OC-SVM algorithm slightly improves in some cases. However, as previously stated, these features are currently not sent in any of the analyzed services in Barcelona. Besides, sending extra features can be detrimental for the network nodes and, therefore, the slight increment in the performance is not worth the effort of adding the extra features in FV3.

## 6. Conclusions

One of the main goals of smart city systems is to collect data from the streets, send it to the city central servers and process it in order to improve municipal services, city planning and the operational functions of certain facilities. Nevertheless, street data is normally gathered using WSNs, which can be easily unreliable and prone to attacks. Furthermore, some public administrations outsource the deployment and operation of these networks to external providers, losing a direct security control over their services. Thus, it is important that smart city administrators verify that the information received from service providers is correct and free of attacks.

In this article, we compared diverse techniques to analyze whether the data received from smart city WSNs is the result of the normal operation of the network or whether it contains some type of anomaly. We used real data from the smart city of Barcelona to simulate WSNs and implement typical attacks. Then, using this data, we compared four anomaly detection techniques based on different principles: Mahalanobis distance, local outlier factor, hierarchical clustering and OC-SVM. We used various feature vectors definitions to identify the optimal network status fields that the service providers have to include to effectively detect attacks. We also considered three scenarios with different maximum levels of permitted false positive rates. As a result of this work, we conclude that OC-SVM is the most suitable technique in the smart city scenario of this article. Moreover, we justified that the optimal network status information that should be gathered for proper attack detection must include the sequence number of the application packet and the battery level. Considering the most restrictive case with a permitted false positive rate lower than 5%, our experiments achieved a true positive rate over 75%. This value is at least 56% higher than the rates achieved with any of the other compared techniques.

This study is, as far as we know, the first contribution that allows pointing out attacks from the central perspective of the smart city administrators. However, there are still unresolved research issues to address in the future. For example, it is necessary to provide a methodology to set up the parameters for OC-SVM to adjust the false positive rate closer to the required PFPR and to improve the detection performance against attacks with low affectation (e.g., 30% and 50% selective forwarding). It is also important to develop a methodology to automatically set up the time window size to aggregate the data before fitting the detection algorithms. A procedure is required to divide large networks in areas with a reduced and manageable number of sensors to apply anomaly detection in a scalable manner. Finally, it is also necessary to experiment with other WSN fields that help identify the specific attack in each situation.

## Figures and Tables

**Table 1:** Metrics.

True positive rate (tpr)	
	$\frac{\mathrm{true}\mathrm{positives}}{\mathrm{true}\mathrm{positives}+\mathrm{false}\mathrm{negatives}}$
False positive rate (fpr)	
	$\frac{\mathrm{false}\mathrm{positives}}{\mathrm{false}\mathrm{positives}+\mathrm{true}\mathrm{negatives}}$
F-score	
	$\frac{\mathrm{true}\mathrm{positives}}{\mathrm{true}\mathrm{positives}+(\mathrm{false}\mathrm{negatives}+\mathrm{false}\mathrm{positives})/2}$

**Table 2:** Results sorted by TPR using test dataset (b) with samples of all the attacks.

FV	PFPR	Technique	F-score	FPR	TPR
FV3	very restrictive	ocsvm	0.872	0.033	0.798
FV3	restrictive	ocsvm	0.857	0.033	0.774
FV2	very restrictive	ocsvm	0.853	0.024	0.762
FV2	restrictive	ocsvm	0.853	0.024	0.762
FV3	permissive	ocsvm	0.843	0.030	0.750
FV1	very restrictive	ocsvm	0.6	0.708	0.729
FV1	restrictive	ocsvm	0.599	0.696	0.723
FV2	permissive	ocsvm	0.809	0.024	0.696
FV1	permissive	ocsvm	0.583	0.681	0.690
FV2	permissive	hierarchical clustering	0.665	0.211	0.552
FV2	permissive	mahalanobis	0.670	0.149	0.542
FV2	restrictive	mahalanobis	0.655	0.098	0.511
FV2	permissive	lofactor	0.641	0.149	0.507
FV3	permissive	hierarchical clustering	0.616	0.220	0.495
FV2	very restrictive	mahalanobis	0.645	0.048	0.487
FV3	permissive	mahalanobis	0.621	0.149	0.484
FV2	restrictive	lofactor	0.631	0.098	0.484
FV3	restrictive	mahalanobis	0.598	0.098	0.448
FV2	very restrictive	lofactor	0.601	0.048	0.44
FV3	permissive	lofactor	0.569	0.149	0.428
FV3	restrictive	hierarchical clustering	0.545	0.140	0.401
FV3	restrictive	lofactor	0.547	0.098	0.395
FV3	very restrictive	mahalanobis	0.535	0.048	0.374
FV2	restrictive	hierarchical clustering	0.517	0.098	0.366
FV3	very restrictive	lofactor	0.514	0.048	0.354
FV1	permissive	hierarchical clustering	0.394	0.158	0.265
FV3	very restrictive	hierarchical clustering	0.340	0.054	0.210
FV2	very restrictive	hierarchical clustering	0.311	0.071	0.191
FV1	restrictive	hierarchical clustering	0.258	0.101	0.156
FV1	permissive	lofactor	0.251	0.149	0.154
FV1	restrictive	lofactor	0.195	0.098	0.113
FV1	permissive	mahalanobis	0.124	0.149	0.071
FV1	very restrictive	hierarchical clustering	0.122	0.057	0.067
FV1	very restrictive	lofactor	0.117	0.048	0.064
FV1	restrictive	mahalanobis	0.112	0.098	0.062
FV1	very restrictive	mahalanobis	0.046	0.048	0.024

**Table 3:** Results of several cases exceeding the PFPR. Cases where PFPR<FPR are highlighted.

FV	Attack	PFPR	Technique	F-score	FPR	TPR
FV2	Selective forwarding 30%	very restrictive	ocsvm	0.811	0.117	0.762
FV2	Selective forwarding 30%	very restrictive	lofactor	0.218	0.048	0.125
FV2	Selective forwarding 30%	very restrictive	mahalanobis	0.598	0.048	0.437
FV2	Selective forwarding 30%	very restrictive	hierarchical clustering	0.003	0.071	0.002
FV2	Selective forwarding 50%	very restrictive	ocsvm	0.82	0.054	0.732
FV2	Selective forwarding 50%	very restrictive	lofactor	0.502	0.048	0.343
FV2	Selective forwarding 50%	very restrictive	mahalanobis	0.609	0.048	0.449
FV2	Selective forwarding 50%	very restrictive	hierarchical clustering	0.003	0.071	0.002
FV2	Selective forwarding 30%	restrictive	ocsvm	0.811	0.117	0.762
FV2	Selective forwarding 30%	restrictive	lofactor	0.348	0.098	0.221
FV2	Selective forwarding 30%	restrictive	mahalanobis	0.613	0.098	0.464
FV2	Selective forwarding 30%	restrictive	hierarchical clustering	0.111	0.098	0.062

**Table 4:** Mean of the standard deviation of all the features of the training dataset (a) and the test dataset (b) with all the attacks for each feature vector definition.

FV	Dataset	Std. Mean
FV1	training dataset (a)	0.48
FV1	test dataset (b)	0.45
FV2	training dataset (a)	0.39
FV2	test dataset (b)	0.60
FV3	training dataset (a)	0.57
FV3	test dataset (b)	0.79
